# *Thymus mastichina* L. essential oils from Murcia (Spain): Composition and antioxidant, antienzymatic and antimicrobial bioactivities

**DOI:** 10.1371/journal.pone.0190790

**Published:** 2018-01-05

**Authors:** Ana-Belen Cutillas, Alejandro Carrasco, Ramiro Martinez-Gutierrez, Virginia Tomas, Jose Tudela

**Affiliations:** 1 GENZ-Group of research on Enzymology, Department of Biochemistry and Molecular Biology-A, Regional Campus of International Excellence "Campus Mare Nostrum", University of Murcia, Murcia, Spain; 2 Novozymes Spain S.A., Madrid, Spain; 3 Department of Analytical Chemistry, University of Murcia, Murcia, Spain; Institute of medical research and medicinal plant studies, CAMEROON

## Abstract

The compositions of essential oils (EOs) from Spanish marjoram (*Thymus mastichina* L.) grown in several bioclimatic zones of Murcia (SE Spain) were studied to determine their absolute and relative concentrations using gas chromatography-mass spectrometry. 1,8-Cineole and linalool were the main components, followed by α-pinene, β-pinene and α-terpineol. (–)-Linalool, (+)-α-terpineol and (+)-α-pinene were the most abundant enantiomers. When the antioxidant capacities of *T*. *mastichina* EOs and their compounds were measured by five methods, EOs and linalool, linalyl acetate, α-terpinene and γ-terpinene, among others, showed antioxidant activities. All four *T*. *mastichina* EOs inhibited both lipoxygenase and acetylcholinesterase activities, and they might be useful for further research into inflammatory and Alzheimer diseases. Bornyl acetate and limonene showed the highest lipoxygenase inhibition and 1,8-cineole was the best acetylcholinesterase inhibitor. Moreover, these EOs inhibited the growth of *Escherichia coli*, *Staphylococcus aureus* and *Candida albicans* due to the contribution of their individual compounds. The results underline the potential use of these EOs in manufactured products, such as foodstuff, cosmetics and pharmaceuticals.

## Introduction

*Thymus mastichina* L., an endemic species of the Iberian Peninsula, is commonly known as Spanish marjoram. It belongs to the *Lamiaceae* family, with leaves arranged in opposite pairs and small zygomorphic and bilabiate flowers [1]. It is an aromatic plant, whose essential oil (EO) consists of a complex mixture of volatile terpenes. Its chemical composition depends on the plant species, culture and environmental conditions [2]. Previous studies from Portugal and other regions of Spain have reported the composition of *T*. *mastichina* EOs (TmEOs) in the form of the relative concentrations of their volatile compounds [2–11]. However, the absolute concentrations of these compounds have not been determined [2–11], although this information would be useful for applications that require knowing the exact quantity of each compound and for detecting solvent dilutions in commercial EOs. Moreover, no chiral characterization of above mentioned TmEOs has been reported [2–11]. However, such data are important for quality assurance, since they help to assess either the genuineness or possible adulteration of the EOs, as well as their origin [12]. Furthermore, the information is required to use TmEOs as a natural source of pure enantiomers [13].

Oxidative processes are involved in several human diseases, such as cancer, atherosclerosis and rheumatoid arthritis [14–18]. In the case of food products, oxidation and microorganism growth are the main cause of food spoilage and foodborne illnesses. Synthetic preservatives are widely used to combat such threats, but their use is continually in the spotlight due to their questionable safety [4]. For this reason, natural preservatives are being actively sought and, in this context, several plant EOs have been reported as being natural antioxidants and antimicrobials [19–23].

Lipoxygenase (LOX) is an enzyme related to inflammatory processes. It oxidizes unsaturated fatty acids with a cis, cis-1,4-pentadiene structure producing conjugated unsaturated fatty acid hydroperoxides and leukotrienes [24]. Its inhibition leads to the reduction of the inflammatory/allergic response, and tumoral and Alzheimer’s disorders [24–26]. Acetylcholinesterase (AChE) is an enzyme that hydrolyses the acetylcholine and other choline esters that function as neurotransmitters in chemical synapses. Their inhibitors increase the neurotransmitter action, and so they are used as insecticides [27] and in the treatment of cancer [28] and Alzheimer's disease [29, 30]. Some studies about TmEO from Portugal reported the inhibition of LOX and AChE [31, 32].

The objective of this work was to make a thorough characterization of TmEOs from Murcia (south-eastern Spain) for the first time, in order to assess possible biotechnological applications of the same. Absolute and relative concentrations of the volatile compounds of these TmEOs were determined using gas chromatography with mass spectrometry detector (GC-MS). This method, with an enantioselective column (EsGC-MS), was used to determine the proportions of the relevant chiral compounds of these TmEOs for the first time in this species. Important bioactivities were studied for these TmEOs, and their main commercially available pure compounds. For this, five complementary methods were applied to evaluate the antioxidant capacity. The inhibition of LOX and AChE was also reported. The antimicrobial effect of these TmEOs and their main relevant biomolecules against *Pseudomonas aeruginosa*, *Escherichia coli*, *Staphylococcus aureus* and *Candida albicans* was studied. Composition-bioactivity relationships between these EOs from Spanish marjoram and their main volatile compounds were established and possible biotechnological applications are proposed.

## Materials and methods

### Plant material

TmEOs were obtained from aerial parts of the cultivated plants grown in Murcia (Spain), collected during the flowering phase (July 2014). The cultivated plants were collected in the farm of Esencias Martinez Lozano S.A. We confirm that the owner of the land gave permission to conduct the study on his farm. Three plant samples for each harvested locality, during the same day, were collected, mixed and ground with a hammer mill to obtain a uniform mixture of plant powder (≤ 1 mm particle size). Then, three hydrodistillation processes were carried out. using 300 g of plant powder in a Clevenger-type apparatus for 3 hours, after collection to avoid the loss and/or the breakdown of volatile biomolecules. EOs were dried over anhydrous sodium sulfate and stored at 4°C until use. TmEO-1 and -3 were obtained from plants grown in the Upper Meso-Mediterranean bioclimatic zone (Caravaca de la Cruz), TmEO-2 from plants grown in Lower Meso-Mediterranean bioclimatic zone (Lorca) and TmEO-4 from plants grown in Supra-Mediterranean bioclimatic zone (Moratalla) [33]. Plant species were identified in the Plant Biology Department of Murcia University by Dr. Pedro Sanchez-Gomez. The voucher specimens are stored in the Department of Biochemistry and Molecular Biology-A (BMBA160620, BMBA160621, BMBA160622, BMBA160623 for TmEO-1, -2, -3 and -4, respectively).

### Reagents and solvents

The chemical compounds used for the antioxidant assays, the reagents for the LOX and AChE inhibition assays and reference antibacterial and antifungal compounds were purchased from Sigma-Aldrich Spain. All compounds were of analytical grade (purity higher than 95%). All culture media were acquired from VWR Chemicals Spain: Mueller-Hinton agar (MHA), Mueller-Hinton broth (MHB), Roswell Park Memorial Institute medium (RPMI-1640), Sabouraud dextrose agar (SDA), tryptic soy broth (TSB) and yeast peptone dextrose (YPD).

Solvents of analytic grade and buffers were purchased from Merck (Madrid, Spain). Type I (18 MΩ cm) deionized water (MilliQ-Reference, Millipore, Madrid, Spain) was used in this work.

### Fast gas chromatography mass-spectrometry (FGC-MS)

The analyses of TmEOs were performed using an Agilent GC7890 chromatograph, coupled with an Agilent MS5975 mass spectrometer detector with electronic impact ionization and single quadrupole. The sandwich injections (0.2 μL air, 0.2 μL isooctane, 0.2 μL air, 0.3 μL sample and 0.2 μL air, described from plunger to needle) were made using a Gerstel automatic multi-purpose sampler MPS-2XT. The chromatography was performed in a low bleed capillary fused-silica column, SLB-5ms from Supelco (15 m length x 0.1 mm internal diameter x 0.1 μm film thickness) with hydrogen as carrier gas (0.8 mL/min) which generates a head pressure of 46.345 psi. This carrier gas was produced with an electrolytic Parker-Domnik-Hunter generator.

The injection conditions were as follows: septum purge 3 mL/min, split ratio 100:1 and injector temperature 300°C. GC oven temperature was kept at 60°C and programmed to raise up to 300°C as follows: to 92°C at a rate 15°C/min, to 96°C at a rate of 1°C/min, to 108°C at a rate of 20°C/min and kept constant for 0.5 min, to 120°C at a rate of 5°C/min, to 160°C at a rate of 20°C/min, to 170°C at a rate of 5°C/min and to 300°C at a rate of 30°C/min, kept constant at 300°C for 0.5 min.

MS was adjusted to the following conditions: electron ionization energy 70 eV, electron-multiplier voltage 1129, acquisition mass range 30–300 m/z, 21.035 scans·s^−1^, transfer line temperature 280°C, ion source temperature 230°C, MS quadrupole temperature 150°C.

Compounds were identified by comparison of their retention times and the mass spectra of commercially available pure standards (S1 Fig) and the NIST 08 and Wiley 7 spectral databases. The TmEO-1 chromatogram is shown in the S2 Fig with the major compounds identified. The quantitative determination was made by means of calibration curves of each commercially available component described in the TmEOs (S1 Table).

### Enantioselective gas chromatography-mass spectrometry (EsGC-MS)

An Astec Chiraldex B-DM column (30 m length x 0.25 mm internal diameter x 0.12 μm film thickness) from Supelco, made of dimethyl, 2,3-di-O-methyl-6-t-butyl silyl β-cyclodextrin, non-bonded to fused silica column, was installed in the previously described device. The injections were similar to the one previously described but, in this case, 0.5 μL of sample was injected. The injector and transfer line temperatures were 200°C. The column temperature was programmed to increase from 35°C to 170°C at a rate of 4°C/min and decrease to 35°C at a rate of 15°C/min. Hydrogen was used as carrier gas (constant flow of 2.5 mL/min, 8 psi starting column head pressure).

To identify both enantiomers, the retention times and the mass spectra of commercially available pure standards were compared with those of the TmEO compounds, and confirmed with the NIST and Wiley spectral data bases. The chromatogram obtained with TmEO-2 is shown in S3 Fig.

### Antioxidant capacity

Five antioxidant methods were performed with TmEOs and their main individual compounds in triplicate, because the antioxidant activity may occur via scavenging different radicals and chelating metal ions [6, 7, 34]. All measurements were made at the end-point of the reaction, except in the ORAC method where kinetic measures were carried out (S4 Fig). The oxygen radical absorbance capacity (ORAC) assay was carried out as described by Ou, Hampsch-Woodill [35] to measure the activity of TmEOs and compounds against peroxyl radical (ROO·). The results were expressed in trolox equivalent antioxidant capacity (TEAC) units (mg trolox equivalent (TE)/g TmEO). The 2,2'-azino-bis(3-ethylbenzothiazoline-6-sulphonic acid) (ABTS) antioxidant method measures the scavenging ability against ABTS radical cation (ABTS·^+^) [36] reported using TEAC units (mg TE/g TmEO). The 2,2-diphenyl-1-picrylhydrazyl (DPPH) method [37] uses the stable free radical DPPH to measure the scavenging capacity of antioxidants towards it, and the results are given in TEAC units (mg TE/kg TmEO). Both ABTS (strong oxidant) and DPPH (weak oxidant) are nitrogen radicals broadly used to determine the antioxidant capacity. The thiobarbituric acid reactive substances (TBARS) method was used to measure the potential antioxidant capacity of TmEOs by decreasing the oxidation of polyunsaturated fatty acids, using soybean lecithin homogenate as lipid-rich media [38]. The results were expressed in mg butylhydroxytoluene equivalents (BHTE)/g TmEO. The chelating power (ChP) method measured the ability of the tested TmEOs to chelate Fe^2+^ ion, following the method of Miguel, Cruz [39]. Ethylenediaminetetraacetic acid (EDTA) was used as positive control and the results were expressed in mg EDTA equivalents (EDTAE)/g TmEO.

### Enzyme inhibition activity

A lipoxidase preparation from *Glycine max* (soybean) (LOX) was acquired from Sigma-Aldrich Spain. LOX inhibitory activity was determined as previously reported [40]. This assay was carried out on a double beam PerkinElmer Lambda 35 spectrophotometer with the UV-Winlab software, at 25°C. This method measures the absorption at 234 nm of the hydroperoxyde conjugated dienes (ε_234_ = 25000 M^-1^ cm^-1^), which are generated from the oxidation of linoleic acid in the presence of oxygen and LOX. Nordihydroguaiaretic acid was used as standard inhibitor.

Cholinesterase acetyl type VI-S (AChE) from *Electrophorus electricus* was purchased from Sigma-Aldrich Spain. AChE inhibitory activity was measured according to Ellman’s method [41]. AChE hydrolyzes acetylthiocholine to acetate and thiocholine, which reacts with 5,5'-Dithiobis(2-nitrobenzoic acid) (DTNB) producing a coloured compound with absorbance at 412 nm. The reaction was measured for 10 min at 25°C, using a 96-well microplate reader. Galantamine hydrobromide was used as reference inhibitor.

In these antienzymatic assays, the degree of inhibition (DI) was calculated using Eq 1:

$$DI\;(\%)=\frac{\nu_{0-}v_{i}}{v_{0}}x100$$ (1)

where *v*_0_ and *v*_*i*_ are the steady state rates in the absence and presence of inhibitor, respectively. The inhibitions of LOX were reported as DI at 150 μg/mL, which is the maximum concentration of TmEO that could be used due to its limit of solubility. However, AChE inhibition could be expressed as IC_50_. To calculate the IC_50_ values, data of DI (%) of seven different concentrations were plotted and fitted by non-linear regression according to Eq 2 using Sigma Plot software [42] (S5 Fig).

$$DI\;(\%)=\frac{DImax\;{\left[I\right]}_{0}}{{IC}_{50}+\;{\left[I\right]}_{0}}$$ (2)

All TmEOs and their compounds were analyzed in triplicate. The inhibition of individual compounds was expressed as IC_50_ or DI, depending on their inhibition capacities and solubilities.

### Antimicrobial activity

#### Microorganisms and culture conditions

The following test microorganisms used in this work were acquired from Sigma-Aldrich: *P*. *aeruginosa* ATCC 9027, *E*. *coli* ATCC 8739, *S*. *aureus* ATCC 6538 and *C*. *albicans* ATCC 10231. The stock cultures were preserved in TSB or YPD with 15% glycerol, for bacteria and yeast cells, respectively, at -80°C.

#### Determination of minimum inhibitory concentrations (MIC) and minimum bactericidal (MBC) or fungicidal (MFC) concentrations

MIC were determined using the microdilution method, according to the M07-A10 [43] standard for bacteria and the M27-A3 [44] for *Candida*. Two-fold dilutions of TmEOs were prepared to obtain a final concentration range of 0.2–18.8 mg/mL with 0.5% Tween^®^80 and 2.5% DMSO. Most compounds were also tested to evaluate their antimicrobial activity in the concentration range of 0.12–15 mmol/L. The final strain concentration was 5 x 10^5^ CFU/mL in MHB for bacteria and 0.5–2.5 x 10^3^ in RPMI-1640 for yeast. These plates were incubated for 24 h for bacteria and 48 h for the yeast, both at 35 ± 1°C, under aerobic conditions on a plate shaker at 100 rpm. Streptomycin (0.06–8 μg/mL) and fluconazole (0.13–16 μg/mL) were used as reference antibacterial and antifungal compound, respectively. The negative and positive controls were made to test that all solutions were sterile and that 0.5% Tween^®^80 and 2.5% DMSO, used for emulsifying the TmEOs, did not show any antibacterial activity. MIC was defined as the lowest concentration of EO with no visible growth of microorganisms, at the end of the incubation period. Then, 100 μl of each well without growth in the MIC assay were spread on MHA (bacteria) or SDA (yeast) and incubated for 24 h at 35 ± 1°C to determinate the MBC or MFC. The MBC or MFC was defined as the lowest EO concentration in which microorganisms failed to grow in broth and on agar. All determinations were carried out in triplicate.

### Statistical analysis

The statistical analyses of data were made using both univariate and multivariate methods [45]. Data were recorded as mean ± standard deviation (SD) of at least triplicate determinations. Data values of 0.0 in the tables mean values lower than 0.05 units. Data quality was analyzed by ANOVA and means were confronted using Tukey’s (HSD) test, considering differences to be significant at p < 0.05, represented by different letters next to numerical values in tables. To determine similarity between TmEOs, Principal Component Analysis (PCA) and agglomerative hierarchical clustering (AHC) based on Euclidean distance were performed. Statistical analyses were conducted using Statistica software (software.dell.com).

## Results and discussion

### FGC-MS study

#### Experimental data

The obtained yields of the TmEO distillation process ranged from 1.8 to 2.6% (v/w). All the identified compounds are detailed in Table 1, where the composition is expressed as percentage of the total area (> 98%) for all compounds, and absolute concentration for commercially available compounds (> 95%). The two major compounds in these TmEOs are 1,8-cineole and linalool, with concentrations that varied from 38.8 to 74.0% for 1,8-cineole and from 2.2 to 42.7% for linalool. 1,8-Cineole was the major compound in TmEO-1, -2 and -3, whereas TmEO-4 had linalool as the most abundant compound. α-Pinene, β-pinene and α-terpineol were also present in relatively high concentrations. Oxygenated monoterpenes were the major group, mainly due to the high concentrations of 1,8-cineole and linalool, as reported in studies from other countries [2, 5–9, 11].

**Table 1:** Fast gas chromatography determination of TmEO compounds.

N^a^	LRI^b^	LRI^c^	Compound	Qualifying and quantitation ions^d^ (m/z)	TmEO-1		TmEO-2		TmEO-3		TmEO-4		IM
					Concentration (mmol/L ± SD)	Area (% ± SD)	Concentration (mmol/L ± SD)	Area (% ± SD)	Concentration (mmol/L ± SD)	Area (% ± SD)	Concentration (mmol/L ± SD)	Area (% ± SD)	
1	928	927	α-Thujene	77, 91, 93, 136		0.1f±0.0		0.1e±0.0		0.1e±0.0		0.1g±0.0	1,2
2	930	936	α-Pinene	77, 91, **93**, 121	109.4c±0.2	2.3g±0.0	128.0a±0.3	2.9e±0.0	112.5b±0.8	2.6f±0.0	65.6d±0.5	1.4h±0.0	1,2,3
3	943	954	Camphene	79, **93**, 121, 136	42.0a±0.4	0.6f±0.0	31.2c±0.2	0.5g±0.0	40.7b±0.7	0.6e±0.0	10.3d±0.2	0.1h±0.0	1,2,3
4	964	975	Sabinene	77, 91, **93**, 136	103.8c±0.4	1.6g±0.0	117.4a±0.7	1.9e±0.0	105.1b±0.3	1.8f±0.0	50.0d±0.3	0.7h±0.0	1,2,3
5	970	983	β-Pinene	69, 79, 91, **93**	169.4c±1.3	2.9g±0.0	205.6a±0.8	3.6e±0.0	176.5b±2.6	3.2f±0.0	104.2d±0.5	1.7h±0.0	1,2,3
6	979	989	Myrcene	41, **69**, 93, 121	63.4a±1.5	0.8e±0.0	62.7a±0.2	0.9e±0.0	54.5b±0.6	0.8f±0.0	35.4c±0.5	0.4g±0.0	1,2,3
7	999	1008	Phellandrene	77, **93**, 119, 136	6.2b±0.2	tr	7.3a±0.6	tr	6.6ab±0.3	tr	2.3c±0.1	tr	1,2,3
8	1008	1017	α-Terpinene	91, **93**, 119, 121	20.5a±0.4	0.3e±0.0	12.9b±0.4	0.2f±0.0	12.4b±0.5	0.2f±0.0	5.0c±0.1	0.1g±0.0	1,2,3
9	1011	1024	p-Cymene	91, 117, **119**, 121	15.4d±0.3	0.4g±0.0	23.3a±0.5	0.7e±0.0	18.9c±0.4	0.6f±0.0	20.9b±0.1	0.6f±0.0	1,2,3
10	1020	1030	Limonene	67, **68**, 79, 93	110.0b±1.3	2.2e±0.0	220.1a±9.3	1.5g±0.2	228.7a±5.2	1.9f±0.1	47.7c±0.4	0.9h±0.0	1,2,3
11	1023	1035	1,8-Cineole	43, 81, **93**, 108	2297.4c±41.1	55.7g±0.2	2742.8a±39.0	74.0e±0.2	2585.8b±2.2	61.6f±0.0	1631.1d±29.2	38.8h±0.1	1,2,3
12	1034	1042	E-β-Ocimene	79, 91, 93, 121,		1.4e±0.0		0.3h±0.0		1.1f±0.0		0.4g±0.0	1,2
13	1053	1056	γ-Terpinene	77, 91, **93**, 119	32.9a±0.4	0.5e±0.0	19.5b±0.5	0.4f±0.0	19.5b±0.3	0.4f±0.0	9.6c±0.0	0.2g±0.0	1,2,3
14	1070	1070	Sabinene hydrate	77, 91, **93**, 121	10.0c±0.3	0.2f±0.0	12.2b±0.6	0.1g±0.0	12.8b±0.4	0.2f±0.0	21.5a±0.7	0.6e±0.0	1,2,3
15	1080	1087	Terpinolene	91, **93**, 121, 136	10.8a±0.2	0.1e±0.0	8.2b±0.3	0.1f±0.0	8.6b±0.2	0.1ef±0.0	5.0c±0.0	0.1g±0.0	1,2,3
16	1081	1103	Linalool	41, 67, **69**, 93	1168.3b±34.6	18.5f±0.1	137.5d±4.1	2.2h±0.0	901.4c±22.2	13.3g±0.0	2357.9a±36.4	42.7e±0.1	1,2,3
17	1089	1105	Hotrienol	71, 82, 91, 119		0.5f±0.0		0.1h±0.0		0.3g±0.0		1.1e±0.1	1,2
18	1148	1145	Camphor	81, **95**, 108, 152	32.3a±0.2	0.2e±0.0	18.1b±0.6	0.1f±0.0	31.2a±0.3	0.2e±0.0	9.4c±0.7	tr	1,2,3
19	1162	1168	δ-Terpineol	41, 59, 79, 93		0.9f±0.0		1.0e±0.0		0.9g±0.0		0.8h±0.0	1,2
20	1174	1171	Borneol	79, 93, **95**, 110	51.9a±1.3	0.9e±0.0	40.2c±0.4	0.6f±0.0	48.7b±1.1	0.8e±0.0	14.0c±0.3	0.1g±0.0	1,2,3
21	1176	1179	Terpinen-4-ol	71, 86, **93**, 111	45.1a±0.4	0.9e±0.0	39.0b±1.0	0.8f±0.0	40.5b±1.0	0.9f±0.0	35.7c±0.5	0.7g±0.0	1,2,3
22	1192	1197	α-Terpineol	59, 67, **93**, 121	181.7a±2.5	3.1e±0.0	170.2b±0.8	3.1e±0.0	155.1c±2.7	2.8f±0.0	182.9a±1.6	3.1e±0.0	1,2,3
23	1215	1227	Nerol	41, 69, 79, 93		0.1f±0.0		0.1f±0.0		tr		0.1e±0.0	1,2
24	1237	1253	Linalyl acetate	41, 69, **93**, 80	59.2b±0.6	1.0f±0.0	46.5c±0.6	0.8g±0.0	43.4d±0.5	0.7h±0.0	104.0a±0.9	1.7e±0.0	1,2,3
25	1238	1256	Geraniol	41, **69**, 93, 123	14.0b±0.7	0.2f±0.0	9.2c±0.6	0.1g±0.0	9.7c±0.4	0.1g±0.0	20.0a±0.4	0.3e±0.0	1,2,3
26	1285	1284	Bornyl acetate	79, **93**, 95, 121	13.9a±0.4	0.2e±0.0	8.9c±0.2	0.1f±0.0	13.2b±0.3	0.2e±0.0	1.7d±0.0	tr	1,2,3
27	1342	1312	Neryl acetate	79, 93, 107, 136		0.2g±0.0		0.4e±0.0		0.3f±0.0			1,2
28	1350	1345	α-Terpinyl acetate	67, 68, **93**, 121	47.0a±0.1	0.8g±0.0	43.2b±0.5	1.2e±0.0	38.1c±0.5	1.1f±0.0	4.5d±0.0	0.1h±0.0	1,2,3
29	1360	1378	Geranyl acetate	41, 69, 93, 121		0.3e±0.0		0.1g±0.0		0.2f±0.0		0.2f±0.0	1,2
30	1412	1403	α-Gurjunene	105, 119, 161, 204		tr		tr		0.1e ± 0.0		tr	1,2
31	1421	1419	E-β-Caryophyllene	**41**, 91, 93, 133	108.7a ± 1.5	0.8f ± 0.0	51.9c ± 0.2	0.3h ± 0.0	111.0a ± 2.2	0.8e ± 0.0	97.3b ± 2.2	0.7g ± 0.0	1,2,3
32	1454	1459	α-Humulene	80, **93**, 121, 204	2.1b ± 0.0	tr			2.3a ± 0.0	0.1e ± 0.0	1.9c ± 0.1	tr	1,2,3
33	1459	1463	Aromadendrene	91, 105, 161, 189		0.1e ± 0.0		0.1f ± 0.0		0.1e ± 0.0		0.1f ± 0.0	1,2
34	1471	1481	γ-Muurolene	105, 119, 161, 204		0.1e ± 0.0		tr				0.1f ± 0.0	1,2
35	1473	1490	Viridiflorene	91, **105**, 107, 161	13.0a ± 0.9	tr	9.4b ± 0.3	0.1e ± 0.0	10.5ab ± 0.6	tr	11.4ab ± 1.7	0.1e ± 0.0	1,2,3
36	1476	1494	γ-Gurjunene	91, 105, 161, 204		0.3e ± 0.0		0.1f ± 0.0		0.2e ± 0.0		0.1f ± 0.0	1,2
37	1494	1497	α-Muurolene	91, 93, 119, 161		tr		tr		tr		tr	1,2
38	1500	1508	β-Bisabolene	41, 69, 93, 204		tr		tr		tr		tr	1,2
39	1507	1511	γ-Cadinene	91, 105, 119, 161		0.1e ± 0.0		tr		0.1e ± 0.0		tr	1,2
40	1514	1517	δ-Cadinene	91, 119, 134, 161		0.1e ± 0.0		0.1f ± 0.0		0.1e ± 0.0		0.1f ± 0.0	1,2
41	1559	1556	Geranyl butyrate	69, 93, 121, 136		tr		tr		tr		tr	1,2
42	1569	1575	Spathulenol	119, 131, 159, 187		0.1g ± 0.0		tr		0.1f ± 0.0		0.1e ± 0.0	1,2
43	1575	1581	Caryophyllene oxide	41, 79, 91, 109		0.1fg ± 0.0		tr		0.1f ± 0.0		0.1e ± 0.0	1,2
44	1594	1594	Viridiflorol	43, 109, 161, 204		0.1ef ± 0.0		0.1ef ± 0.0		0.1f ± 0.0		0.1e ± 0.0	1,2
			Alcohol			25.48		8.18		19.57		49.70	
			Ketone			0.17		0.10		0.18		0.00	
			Ester			2.66		2.65		2.61		2.10	
			Ether			55.72		73.25		61.65		38.87	
			Monoterpene hydrocarbons			13.31		13.05		13.39		6.71	
			Oxygenated monoterpenes			83.71		83.88		83.69		90.23	
			Sesquiterpene hydrocarbons			1.52		1.75		1.55		1.12	
			Oxygenated sesquiterpenes			0.32		0.29		0.32		0.44	
			Total terpene hydrocarbons			14.83		14.79		14.94		7.83	
			Total oxygenated terpenes			84.03		84.17		84.01		90.67	

^a^Reference number for statistical PCA graphs.

^b^Linear Retention Index from data bases NIST 08 & Wiley 7.

^c^Linear Retention Index calculated from the homologous series of n-alkanes (C7-C30).

^d^Ions used for quantitation are in bold. IM = Identification method: 1 = by LRI, 2 = by NIST 08 & Wiley 7, 3 = by comparison with pure compounds. tr = Traces (<0.1%). Different letters next to numerical values, represent significant differences at p < 0.05 resulting from ANOVA plus HSD test.

#### Multivariate statistic PCA

**Fig 1:**
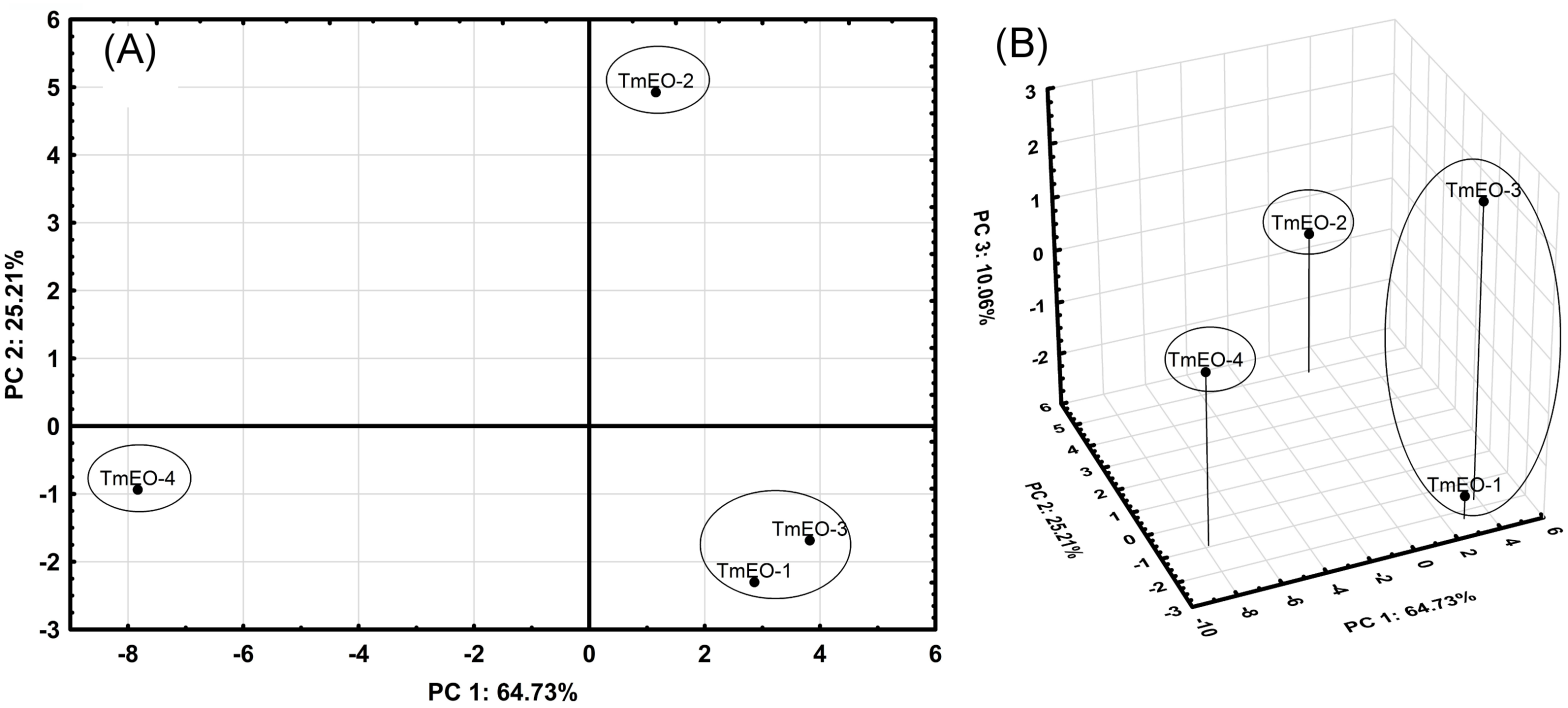
PCA score plots. (A) Score plot of PC2 vs. PC1. (−) tentative two-dimensional clustering. (B) 3D-Score plot of PC3 vs. PC2 and PC1. (−) tentative three-dimensional clustering. The loading plot of PC2 and PC1 (Fig 2) shows the “characteristic” compounds of each cluster. The loadings of compounds are standardized. A high load of a compound indicates that its presence (high or low percentage of the total area) is “characteristic” of that TmEO. TmEO-1 and -3 are characterized by the high proportion of 1,8-cineole (11), as well as the average level proportion of linalool (16). For their part, β-ocimene (12), E-β-caryophyllene (31), γ-gurjunene (36) and γ-cadinene (39) are found in higher percentages in TmEO-1 and -3 than in the other TmEOs. TmEO-2 shows a characteristic high concentration of 1,8-cineole (11), and also of β-pinene (5), and δ-terpineol (19). Characteristic compounds of TmEO-4 are the high proportion of linalool (16), hotrienol (17), linalyl acetate (24) and caryophyllene oxide (43). These qualitative data are useful to explain the quantitative similarities between the clusters considered in the AHC analysis.

**Fig 2:**
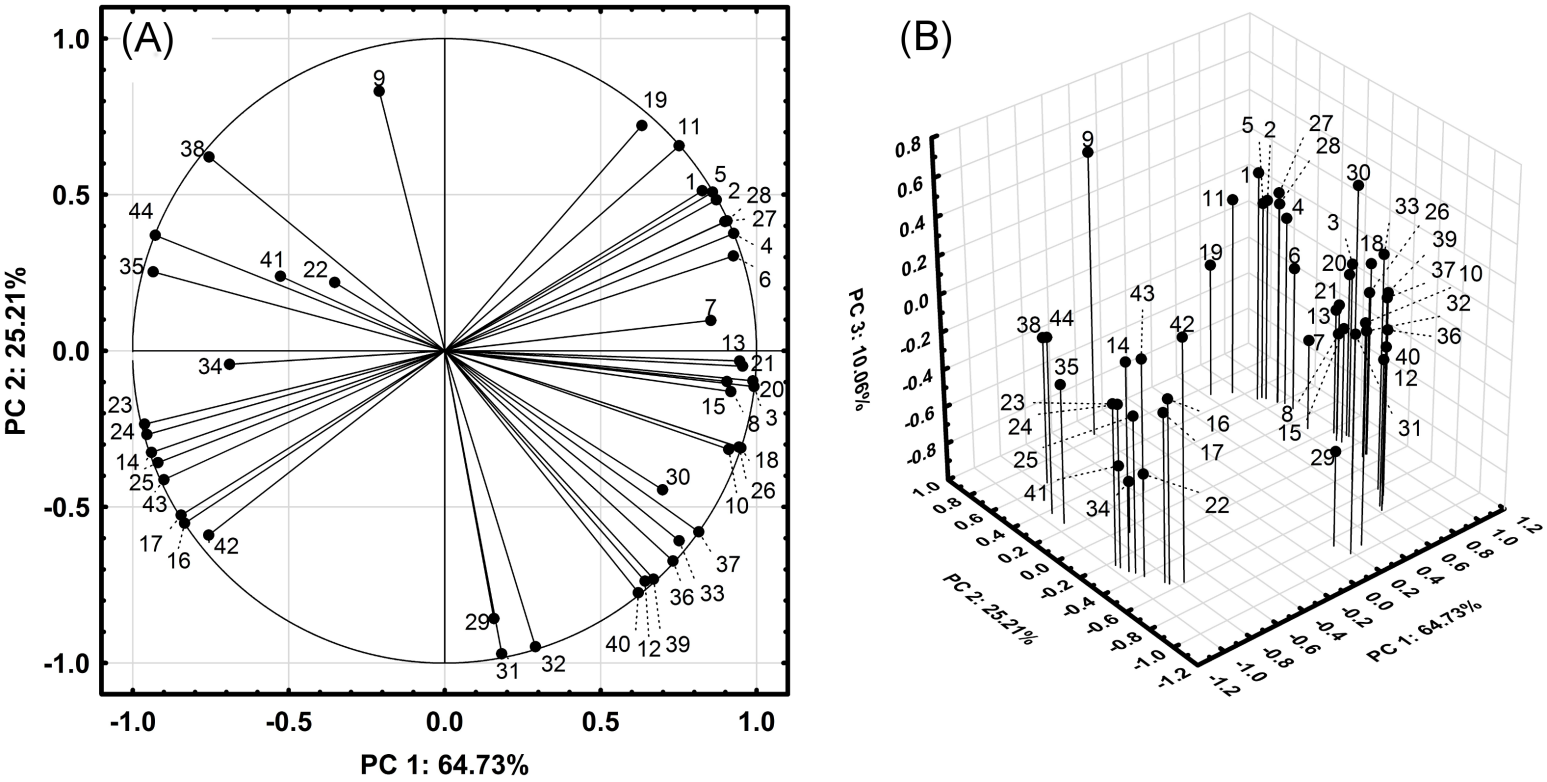
PCA loading plots. (A) Loading plot of PC2 vs. PC1. (B) 3D-loading plot of PC3 vs. PC2 and PC1.

The PCA is based on the covariance matrix between linear combinations of the experimental variables (Table 1) and provides information about the qualitative similarities between EOs (Fig 1) and their characteristic compounds (Fig 2).

The first (PC1), second (PC2) and third (PC3) principal components account for 64.3%, 25.21% and 10.06% of whole variance, respectively. Thus, the cumulative proportion of total variance of these principal components is 100%.

The score plot of PC2 vs. PC1 (Fig 1A) shows three clusters: (1) TmEO-1 and -3; (2) TmEO-2; (3) TmEO-4. TmEO-1 and -3 show higher differences when the PC3 is represented vs. PC2 and PC1 (Fig 1B).

#### Multivariate statistic AHC

**Fig 3:**
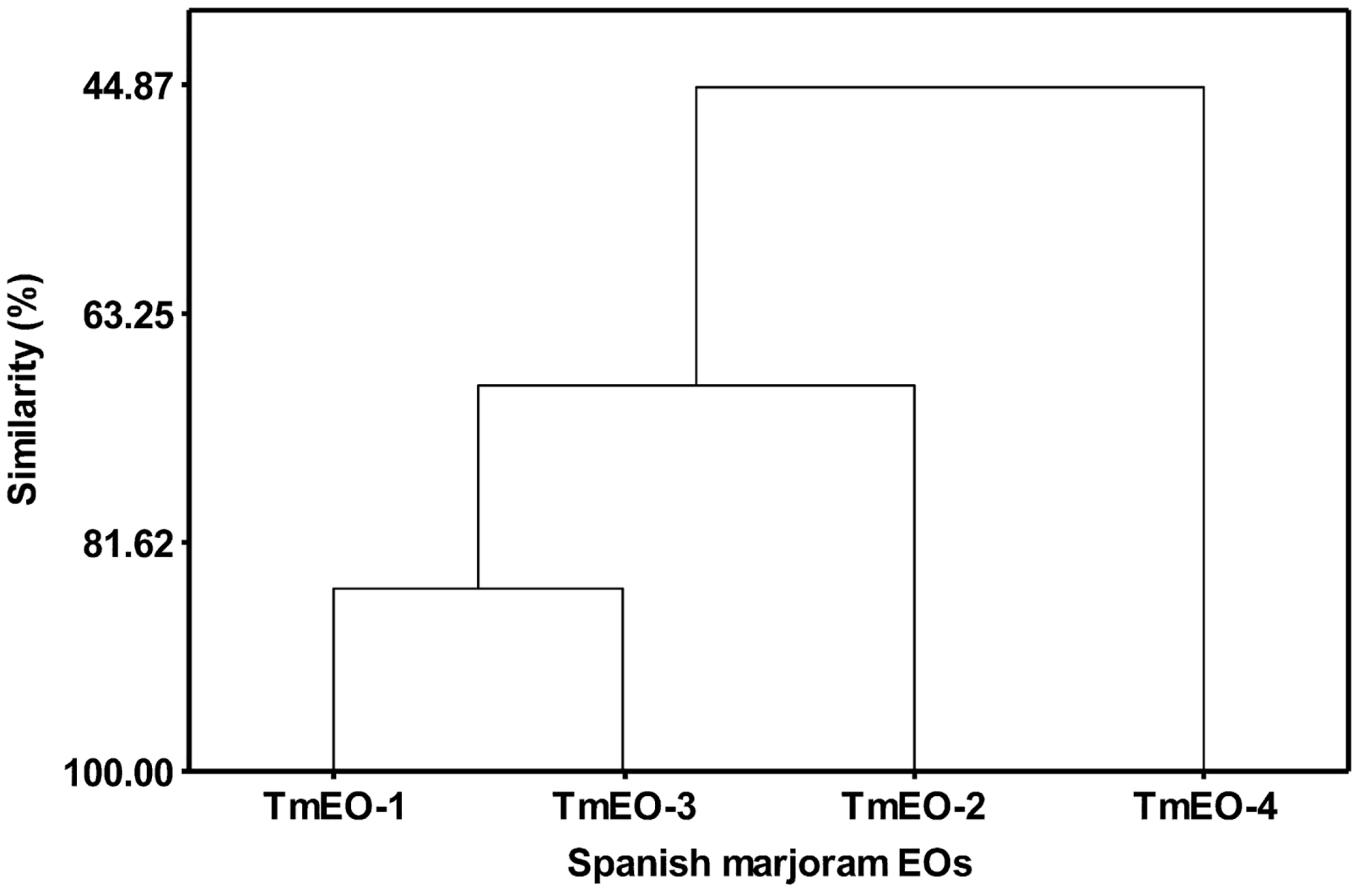
AHC dendrogram. Percentage of similarities between studied TmEOs and clusters.

The dendrogram (Fig 3) represents the agglomerative hierarchical clustering based on Euclidean distance, showing that TmEO-1 and -3 are the most similar (85.2% similarity), clearly different from TmEO-2 (69.0% similarity) and TmEO-4 (44.9% similarity). The consideration of the whole compounds of TmEOs provides quantitative data of similarities between TmEOs, allowing us to identify three clusters (similar to the preliminary estimations in the PCA analysis).

#### Comparison with other regions and countries

To our knowledge, there is only one other study on TmEO from Spain [4], in which 1,8-cineole and, to a much lesser extent, linalool were reported to be the main components. However, the variability of the 1,8-cineole content in these TmEOs was higher than that described in the above study. Among the studies about TmEO obtained from plants grown in Portugal [2, 3, 7–11], one study [3] described a TmEO with 44% of 1,8-cineole and a higher concentration of camphor, borneol, camphene, α-pinene and α-terpineol than in our study. Other studies [2, 8] reported TmEOs with high percentages of 1,8-cineole, but in some cases, it was lower than the percentage of linalool and lower than the percentages described in this study. The composition of TmEOs can show high variability depending on the growing area [2]. Other TmEOs from Portugal [10] showed a TmEO composition with high concentrations of p-cymene, γ-terpinene, thymol and carvacrol. The last two compounds were not found in our TmEOs or in other studies. TmEOs from Italy [5] showed a similar composition to our study, with high concentrations of 1,8-cineole and linalool, followed by α-pinene, β-pinene and α-terpineol. Although all EOs were obtained from the same plant species, their compositions can be strongly influenced by physiological and genetic variations and environmental conditions [34].

### International standard comparative

The compositions of these four TmEOs match the standards established by the International Organization for Standardization [46] (ISO) (Table 2). Only in the case of TmEO-2 was the concentration of linalool and β-caryophyllene slightly lower than the standard range, whereas the proportion of 1,8-cineole was higher than the standard range interval.

**Table 2:** TmEO compositions compared with ISO standards.

Compound	ISO standard^a^		TmEO-1 (%)	TmEO-2 (%)	TmEO-3 (%)	TmEO-4 (%)
	minimum (%)	maximum (%)				
α-Pinene	1.0	4.5	2.3	2.8	2.6	1.4
β-Pinene	2.0	5.0	2.9	3.6	3.2	1.7
Limonene	1.0	6.0	2.2	1.6	1.9	0.9
1,8-Cineole	30.0	68.0	55.7	73.2	61.6	38.8
Linalool	3.0	48.0	18.5	2.1	13.3	42.7
Camphor	0.1	2.0	0.2	0.1	0.2	tr
δ-Terpineol	0.2	2.0	0.9	0.9	0.9	0.8
Borneol	0.1	1.8	0.9	0.6	0.8	0.1
Terpinen-4-ol	0.2	1.2	0.9	0.8	0.9	0.7
Linalyl acetate	0.2	4.0	1.0	0.8	0.7	1.7
β-Caryophyllene	0.5	1.5	0.8	0.3	0.8	0.7
α-Terpineol	1.0	5.0	3.1	3.1	2.8	3.1

^a^ISO standard 4728 for Spanish wild marjoram (*Thymus mastichina* L.) [46].

### EsGC-MS study

In the present study, the enantiomeric distributions of the commercially available compounds of these TmEOs were analyzed (Table 3 and S3 Fig). The (+)-enantiomer predominates in the case of α-pinene, limonene, sabinene hydrate, terpinen-4-ol, α-terpineol, α-terpinyl acetate and aromadendrene. The (–)-enantiomer is the most abundant in the case of camphene, linalool, camphor, bornyl acetate, borneol and β-caryophyllene. The enantiomeric distributions were similar for all compounds in all the TmEOs. The case of (–)-linalool was an exception: in TmEO-1 and -3 its concentration was nearly 90% of the area, whereas in TmEO-2 and -4 the concentration of (–)-linalool was nearly 50% of the area. To our knowledge, no similar enantiomeric determinations have been made for TmEOs.

**Table 3:** Enantiomeric ratios of TmEO compounds^a^.

t_R_		Compound (X)	TmEO-1		TmEO-2		TmEO-3		TmEO-4	
(min)			(+)—[X] (%)	(–)—[X] (%)	(+)—[X] (%)	(–)—[X] (%)	(+)—[X] (%)	(–)—[X] (%)	(+)—[X] (%)	(–)—[X] (%)
(+)—X	(–)—X									
7.58	7.36	α-Pinene	83.7	16.3	87.1	12.9	85.0	15.0	87.1	12.9
8.47	8.24	Camphene	<5.0	>95.0	<5.0	>95.0	<5.0	>95.0	<5.0	>95.0
8.687	8.934	β-Pinene	50.0	50.0	49.9	50.1	50.0	50.0	49.9	50.1
10.27	9.86	Limonene	>95.0	<5.0	>95.0	<5.0	>95.0	<5.0	>95.0	<5.0
14.11	14.36	Sabinene hydrate	>95.0	<5.0	>95.0	<5.0	>95.0	<5.0	>95.0	<5.0
15.6	15.33	Linalool	6.2	93.8	47.4	52.6	6.8	93.2	47.3	52.7
16.26	16.10	Camphor	<5.0	>95.0	<5.0	>95.0	<5.0	>95.0	<5.0	>95.0
17.55	17.80	Bornyl acetate	<5.0	>95.0	<5.0	>95.0	<5.0	>95.0	<5.0	>95.0
18.13	18.29	Terpinen-4-ol	71.2	28.8	72.5	27.5	71.2	28.8	72.5	27.5
19.76	19.31	Borneol	<5.0	>95.0	<5.0	>95.0	<5.0	>95.0	<5.0	>95.0
19.85	19.49	α-Terpineol	65.5	34.5	67.4	32.6	67.7	32.3	67.4	32.6
20.91	22.35	α-Terpinyl acetate	>95.0	<5.0	>95.0	<5.0	>95.0	<5.0	>95.0	<5.0
23.72	23.53	Aromadendrene	>95.0	<5.0	>95.0	<5.0	>95.0	<5.0	>95.0	<5.0
-	22.56	β-Caryophyllene	<5.0	>95.0	<5.0	>95.0	<5.0	>95.0	<5.0	>95.0

^a^SD lower than ± 5%.

### Antioxidant activity

The antioxidant activities of the TmEOs from Murcia and their main individual compounds have been evaluated using several complementary methods, as it is usual for the study of EOs from other plants and countries [6, 7, 34]. The capacities of the TmEOs and their compounds for scavenging of peroxyl radicals (ORAC [35]), strong oxidant nitrogen radicals (ABTS [36]), weak oxidant nitrogen radicals (DPPH [37]), and lipidic peroxyl radicals (TBARS [38]),as well as for chelating oxidant metal ions such as Fe^2+^ (ChP [39]) have been determined. These assays could lead to different and complementary antioxidant activities that will be described and discussed below.

#### ORAC

The antioxidant activity was expressed in TEAC units (mg TE/g TmEO) and is reported in Table 4. The results can be ordered as follows: TmEO-4^ORAC^ > TmEO-1^ORAC^ > TmEO-3^ORAC^ > TmEO-2^ORAC^. The antioxidant activity of individual compounds was assayed (Table 4) to determine which compounds were responsible for these differences between the TmEOs. According to the results, linalool, terpinen-4-ol, α-terpineol, linalyl acetate and β-caryophyllene were the best antioxidant compounds in this assay—the higher the linalool and linalyl acetate concentrations, the higher the antioxidant activity of the TmEOs. Higher antioxidant capacity was observed in a previous study [10] with a TmEO from Portugal, which contained thymol and carvacrol.

**Table 4:** Antioxidant capacity of TmEOs and their main individual compounds^a^.

TmEO/Compound	ORAC (mg TE/g X)	ABTS (mg TE/g X)	DPPH (mg TE/kg X)	TBARS (mg BHTE/g X)	ChP (mg EDTAE/g TmEO)
TmEO-1	485.1b ± 23.8	4.3a ± 0.1	53.5c ± 1.3	1.2a ± 0.2	0.6d ± 0.0
TmEO-2	163.5d ± 8.8	0.9bc ± 0.0	61.3b ± 3.5	0.9a ± 0.2	1.6a ± 0.1
TmEO-3	371.8c ± 15.1	1.0b ± 0.0	62.9b ± 2.3	1.2a ± 0.2	0.8c ± 0.0
TmEO-4	735.1a ± 35.4	0.8c ± 0.1	76.1a ± 3.6	1.0a ± 0.2	1.0b ± 0.0
α-Pinene	N/D	N/D	37.1 ± 3.4	4.2 ± 0.1	35.7 ± 2.4
Camphene	N/D	0.2 ± 0.0	N/D	N/D	3.4 ± 0.3
β-Pinene	50.9 ± 2.9	0.2 ± 0.0	16.3 ± 1.0	7.2 ± 0.5	3.9 ± 0.2
Myrcene	N/D	N/D	N/D	N/D	5.3 ± 0.4
α-Terpinene	N/D	7.3 ± 0.4	504.3 ± 21.6	N/D	133.4 ± 10.0
p-Cymene	N/D	0.2 ± 0.0	N/D	N/D	43.2 ± 3.4
Limonene	244.9 ± 20.6	1.1 ± 0.1	N/D	N/D	12.7 ± 0.8
1,8-Cineole	N/D	N/D	N/D	N/D	2.1 ± 0.1
γ-Terpinene	304.6 ± 17.9	4.6 ± 0.2	607.0 ± 20.9	70.8 ± 6.3	0.7 ± 0.0
Sabinene hydrate	69.3 ± 5.4	0.8 ± 0.0	N/D	20.8 ± 0.9	12.7 ± 1.0
Linalool	536.7 ± 28.2	0.1 ± 0.0	N/D	N/D	183.6 ± 11.1
Camphor	N/D	N/D	N/D	N/D	N/D
Borneol	N/D	N/D	N/D	N/D	N/D
Terpinen-4-ol	601.5 ± 22.0	0.5 ± 0.0	73.8 ± 2.0	11.9 ± 2.5	3.2 ± 0.2
α-Terpineol	523.8 ± 27.4	0.3 ± 0.0	N/D	3.7 ± 0.1	9.3 ± 0.6
Linalyl acetate	255.5 ± 15.9	0.1 ± 0.0	N/D	4.8 ± 0.2	40.8 ± 3.1
Bornyl acetate	N/D	N/D	N/D	N/D	N/D
β-Caryophyllene	483.5 ± 17.7	N/D	N/D	18.6 ± 0.8	10.2 ± 0.7

^a^N/D = Activity lower than 0.05 units at a maximum assay concentration of 100 mmol/L. X = TmEO or compound. Different letters next to numerical values, represent significant differences at p < 0.05 resulting from ANOVA plus HSD test.

#### ABTS

The results of the ABTS method (Table 4) in TEAC units (mg TE/g TmEO) were as follows: TmEO-1^ABTS^ > TmEO-3^ABTS^ ≥ TmEO-2^ABTS^ ≥ TmEO-4^ABTS^. As regards individual compounds (Table 4), α-terpinene and γ-terpinene showed the highest antioxidant activities in this method. TmEO-1 contained a higher concentration of both compounds, which may help explain the different activities of the TmEOs. A TmEO from Portugal [10] showed higher antioxidant activity in this method, probably due to the presence of thymol and carvacrol.

#### DPPH

The DPPH assay gave the following results (Table 4) in TEAC units (mg TE/kg TmEO): TmEO-4^DPPH^ > TmEO-3^DPPH^ ≈ TmEO-2^DPPH^ > TmEO-1^DPPH^. According to this method, α-terpinene and γ-terpinene showed the highest antioxidant activities when the compounds were tested individually. However, TmEO-4 has higher antioxidant activity than the others; hence, some synergistic or antagonistic effects may be occurring between the components [9, 47, 48].

The TmEOs studied here showed higher DPPH scavenging activity than those reported for TmEOs from other regions of Spain [4] and Portugal [3, 6].

#### TBARS

The results obtained after testing this method were as shown in Table 4 (mg BHTE/g TmEO): TmEO-3^TBARS^ ≈ TmEO-1^TBARS^ ≈ TmEO-4^TBARS^ ≈ TmEO-2^TBARS^. Several compounds are effective against lipid oxidation (Table 4), producing similar antioxidant activity to the TmEOs in this method.

Other studies [3, 6–8] with TmEOs from Portugal also measured the antioxidant activity by this method, and reported around 5–30% higher antioxidant activity than in our study. EOs from *Origanum vulgare* or *Thymbra capitata* [6] showed higher scavenging effect with lipid radicals than the EOs of this study, probably due to the presence of thymol and carvacrol in those EOs.

#### Chelating power

The measurement of the TmEO chelating power showed the following results (Table 4) expressed in mg EDTAE/g TmEO: TmEO-2^ChP^ > TmEO-4^ChP^ > TmEO-3^ChP^ > TmEO-1^ChP^. There are several compounds with chelating power (Table 4), α-terpinene and linalool the most active being.

The results reported with this method are similar to those obtained with some TmEOs from Portugal [6]. However, other TmEOs from Portugal [3] did not show chelating activity, even though the concentration assayed was similar to that used in the present study.

### Antienzymatic activity

#### LOX inhibitory activity

The four TmEOs were tested at 150 μg/mL to calculate the DI (%). The results were as follows (Table 5): TmEO-1^LOX^ > TmEO-4^LOX^ ≈ TmEO-2^LOX^ > TmEO-3^LOX^.

**Table 5:** Antienzymatic activity of TmEOs and their individual compounds^a^.

TmEO/Compound	LOX inhibition		AChE inhibition	
	IC_50_ (μg/mL)	DI (%) [μg/mL]	IC_50_ (μg/mL)	DI (%) [μg/mL]
TmEO-1		56.7a ± 1.6 [150]	57.5c ± 2.8	
TmEO-2		46.3b ± 1.0 [150]	71.1b ± 3.1	
TmEO-3		40.8c ± 1.0 [150]	72.3b ± 2.0	
TmEO-4		47.6b ± 1.5 [150]	117.2a ± 5.6	
α-Pinene		N/D	446.1 ± 7.9	
Limonene	116.1 ± 3.3			N/D
1,8-Cineole		30.9 ± 1.1 [514.2]	35.2 ± 1.5	
Linalool	516.0 ± 6.8			N/D
Camphor	417.7 ± 13.0			N/D
Terpinen-4-ol		29.6 ± 1.0 [514.2]		16.6 ± 0.9 [650.0]
α-Terpineol		17.4 ± 0.2 [514.2]		N/D
Bornyl acetate	74.5 ± 2.8			N/D

^a^N/D = Activity not detected.

Similarly to the antioxidant methods, the enzymatic inhibition was explained by reference to the inhibition of the TmEO components. The IC_50_ for bornyl acetate, limonene, camphor and linalool were calculated. However, other compounds did not reach 50% inhibition and so the DI at an equal concentration (514.2 μg/mL) is expressed (Table 5). The IC_50_ obtained with NDGA was 102.6 ± 2.8 μg/mL.

Although linalool is not the best LOX inhibitor, it may contribute to TmEO-4 inhibition due to its high concentration.

The DI found in this study are higher than those previously reported for TmEOs from Portugal [31, 32] (IC_50_ values of 0.7 ± 0.0 mg/mL and 1.1 ± 0.1 mg/mL, respectively). This activity may indicate antioxidant and anti-inflammatory capacity of TmEOs.

#### AChE inhibition

The IC_50_ for AChE inhibition were as follows (μg/mL) (Table 5): TmEO-1^AChE^ < TmEO-2^AChE^ ≈ TmEO-3^AChE^ < TmEO-4^AChE^.

After testing the inhibition of individual compounds, it was concluded that 1,8-cineole is the best AChE inhibitor with an IC_50_ of 35.2 ± 1.5 μg/mL. Moreover, some other compounds can inhibit AChE activity (Table 5). TmEO-1, -2 and -3 had higher amounts of 1,8-cineole than TmEO-4, so that, TmEO-4 showed a higher IC_50_ than the others.

Galantamine was used as standard inhibitor (IC_50_ = 0.16 ± 0.03 μg/mL).

The AChE inhibition of the TmEOs and 1,8-cineole was higher than that reported with EOs from other plant species [49]. TmEO from Portugal [31, 32] showed similar AChE inhibition to that reported in this study. These results support the possible use of TmEOs as aid in the treatment of Alzheimer's disease or in its prevention for people with family precedents.

### Antimicrobial activity

The TmEOs were tested against *P*. *aeruginosa*, *E*. *coli*, *S*. *aureus* and *C*. *albicans* using assay concentrations in the range of 0.2–18.8 mg/mL. *P*. *aeruginosa* could not be inhibited even with the highest tested concentration. The other microorganisms were inhibited with TmEO concentrations in the range of 2.3–9.4 mg/mL (Table 6), showing weak antimicrobial capacities, compared to those of the reference antimicrobials. In the case of *E*. *coli*, the most and least effective TmEOs were TmEO-4 and -2, respectively. Some individual compounds inhibited *E*. *coli* (Table 6); more specifically, the most influential compound in this respect was linalool, due to the high concentration found in TmEO-4. In the case of *S*. *aureus* and *C*. *albicans*, the differences between TmEOs were less pronounced. TmEO-4 produced a higher inhibition of *C*. *albicans* than the other TmEOs due to the high concentration of linalool. Similar MIC value for *Candida* was found previously using TmEO from Portugal [50], however, other studies [51, 52] reported lower antibacterial activities of TmEOs than those found in this study. The EOs from other *Thymus* species showed lower MIC values, probably due to the high content of phenolic compounds, such as thymol and carvacrol [53, 54]. EOs obtained from other plant species showed lower or similar antimicrobial capacities [55, 56].

**Table 6:** Antimicrobial capacity of TmEOs and main individual compounds.

TmEO^a^/Compound^b^	*Escherichia coli*		*Staphylococcus aureus*		*Candida albicans*	
	MIC (mg/mL)	MBC (mg/mL)	MIC (mg/mL)	MBC (mg/mL)	MIC (mg/mL)	MFC (mg/mL)
TmEO-1	4.6	4.6	2.3	4.6	4.6	4.6
TmEO-2	9.4	9.4	4.7	4.7	4.7	4.7
TmEO-3	4.6	4.6	4.6	4.6	4.6	4.6
TmEO-4	2.3	2.3	4.6	4.6	2.3	2.3
α-Pinene	0.5	1.0	2.1	>2.1	0.5	0.5
Camphene	>2.0	>2.0	>2.0	>2.0	>2.0	>2.0
Sabinene	>2.0	>2.0	>2.0	>2.0	>2.0	>2.0
β-Pinene	>2.0	>2.0	>2.0	>2.0	>2.0	>2.0
Myrcene	>2.1	>2.1	>2.1	>2.1	>2.1	>2.1
p-Cymene	1.0	2.0	>2.0	>2.0	0.5	0.5
Limonene	2.0	2.0	0.3	0.3	1.0	1.0
1,8-Cineole	>2.3	>2.3	>2.3	>2.3	>2.3	>2.3
γ-Terpinene	>2.0	>2.0	>2.0	>2.0	>2.0	>2.0
Sabinene hydrate	>2.3	>2.3	2.3	2.3	>2.3	>2.3
Linalool	1.1	2.3	0.6	1.1	2.3	2.3
Borneol	1.1	1.1	0.3	0.3	0.6	0.6
Terpinen-4-ol	2.3	2.3	1.1	2.3	>2.3	>2.3
α-Terpineol	2.4	2.4	0.6	1.1	>2.4	>2.4
Linalyl acetate	>3.0	>3.0	3.0	>3.0	>3.0	>3.0
β-Caryophyllene	>3.1	>3.1	>3.1	>3.1	>3.1	>3.1
Streptomycin sulfate	1.0 x 10^−3^	1.0 x 10^−3^	1.0 x 10^−3^	1.0 x 10^−3^	NT	NT
Fluconazole	NT	NT	NT	NT	4.0 x 10^−3^	4.0 x 10^−3^

^a^NT = Not tested

## Conclusions

This work has deepened our knowledge of four TmEOs from plants cultivated in the province of Murcia (Spain). Their compositions differ especially in the content of linalool and 1,8-cineole, whereas PCA and AHC identified three clusters. The proportions of their main enantiomers have been quantified. The concentrations of linalool, linalyl acetate, α-terpinene and γ-terpinene determined the antioxidant activity of the TmEOs. Lipoxygenase and acetylcholinesterase activities were inhibited at low TmEO concentrations. Moreover, the TmEOs inhibit the growth of *E*. *coli*, *S*. *aureus* and *C*. *albicans* in the range of 2.3–9.4 mg/mL. These results support the potential applications of these TmEOs as natural ingredients in nutracosmeceutical products.

## Supporting information

S1 Fig Mass spectra of 1,8-cineole. Comparison between mass spectra of commercial and natural EO compounds.
(TIF)

S2 Fig Chromatogram of TmEO-1. Main compounds are identified with different numbers.
(TIF)

S3 Fig Enantioselective chromatogram of TmEO-2. The main dextrorotatory (+) and levorotatory (−) enantiomers are identified with different numbers.
(TIF)

S4 Fig Results obtained with TmEO using ORAC method. Fluorescence decay curves corresponding to different concentrations of TmEO-4.
(TIF)

S5 Fig Determination of IC_50_ of TmEOs and 1,8-cineole. Kinetic analysis of enzyme inhibition data using non-linear regression.
(TIF)

S1 Table Calibration curve parameters to determine the absolute volatile concentrations of the TmEOs. (PDF)
