# Supplementary figures and images for: Thymus mastichina L. essential oils from Murcia (Spain): Composition and antioxidant, antienzymatic and antimicrobial bioactivities
Source: PLoS One. 2018 Jan 5;13(1):e0190790. doi: 10.1371/journal.pone.0190790 (PMC5755899; doi:10.1371/journal.pone.0190790)

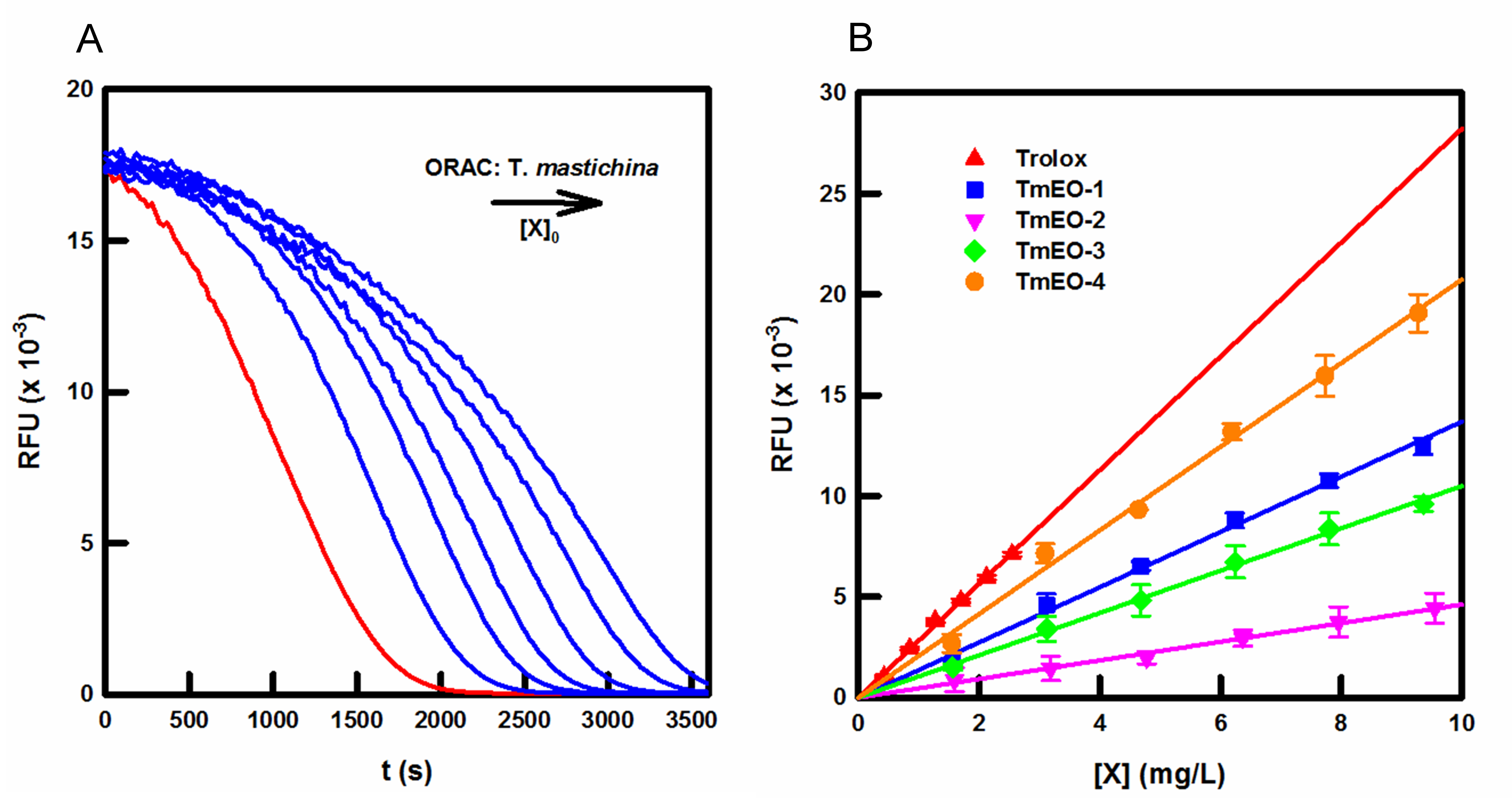

Supplement: S4 Fig — Fluorescence decay curves corresponding to different concentrations of TmEO-4. (TIF) [file pone.0190790.s004.tif]
