# Supplementary material for: Thymus mastichina L. essential oils from Murcia (Spain): Composition and antioxidant, antienzymatic and antimicrobial bioactivities
Source: PLoS One. 2018 Jan 5;13(1):e0190790. doi: 10.1371/journal.pone.0190790 (PMC5755899; doi:10.1371/journal.pone.0190790)
Supplement: S1 Table — (PDF) [file pone.0190790.s006.pdf]

S1 Table. Calibration curve parameters to determine the absolute volatile concentrations of the TmEOs.

Compound	Calibration curve[a]	R^2	Calibration range (mM)	RSD (%)	LOD (mM)	LOQ (mM)	Standard source	Product reference
Nonane	Internal standard (MS 43[b], 57, 71, 85)						SAFC	442694
(-)α-Pinene	y=1.241x-0.010	0.999	16.76-0.25	0.25	0.025	0.076	Fluka	80599
(+)Camphene	y=0.587x-0.002	0.999	18.49-0.28	1.15	0.054	0.165	SAFC	w222909
Sabinene	y=-0.869x+0.027	0.999	16.31-0.61	2.73	0.091	0.277	Extrasynthese	5062 S
(-)β-Pinene	y=0.980x-0.013	0.999	17.16-0.26	0.88	0.026	0.078	Aldrich	112089
Myrcene	y=0.5940x-0.0307	0.998	14.94-0.22	3.25	0.018	0.056	Fluka	64643
(-)Phellandrene	y=0.395x-0.008	0.999	15.87-0.60	2.10	0.106	0.322	Aldrich	77429
α-Terpinene	y=0.573x-0.018	0.999	15.75-0.59	1.72	0.149	0.453	Aldrich	86473
p-Cymene	y=2.580e-0.039	0.999	16.59-0.25	0.08	0.010	0.030	Aldrich	c121452
(+)Limonene	y=0.716x-0.018	0.999	15.92-0.24	1.65	0.039	0.120	Fluka	62118
1,8-Cineole	y=0.356x+0.009	0.999	15.92-0.60	2.70	0.164	0.497	SAFC	w246506
γ-Terpinene	y=0.976x-0.026	0.999	16.46-0.25	0.72	0.020	0.062	Aldrich	223190
Sabinene hydrate	y=0.558x-0.024	0.998	16.10-0.60	0.23	0.102	0.309	Fluka	96573
Terpinolene	y=0.644x-0.009	1.000	15.34-0.92	1.22	0.230	0.699	SAFC	W304603
(-)Linalool	y=0.4378x-0.026	0.997	14.66-0.55	1.53	0.102	0.309	Fluka	74856
Tetradecane	Internal standard (MS 43, 57[b], 71, 85)						SAFC	442708
(+)Camphor	y=0.2375ex-0.038	0.997	16.06-0.96	2.17	0.241	0.733	Alfa Aesar	A10708
(-)Borneol	y=0.3820-0.056	0.993	14.99-0.90	1.51	0.196	0.595	Alfa Aesar	A12684
(-)Terpinen-4-ol	y=0.3517x-0.012	1.000	15.95-0.96	1.47	0.239	0.727	Aldrich	11584
(+)α-Terpineol	y=0.218x+0.001	1.000	16.45-0.99	3.59	0.247	0.750	Fluka	83073
Linalyl acetate	y=0.263x-0.013	0.998	11.97-1.80	3.03	0.449	1.365	SAFC	w263605
Geraniol	y=0.376x-0.023	0.999	15.04-0.90	1.85	0.295	0.896	SAFC	w250716
(-)Bornyl acetate	y=0.380x+0.005	0.999	13.59-0.82	3.25	0.204	0.620	Fluka	45855
(+)α-Terpinyl acetate	y=0.369x-0.009	1.000	11.95-1.79	2.37	0.243	0.740	Aldrich	86487
Hexadecane	Internal standard (MS 41, 43, 57[b], 71)						Fluka	52209
(-)E-β-Caryophyllene	y=0.048x+0.002	0.998	7. 25-1.09	6.07	0.360	1.093	Aldrich	22075
α-Humulene	y=0.731x-0.043	0.999	10.58-0.63	2.32	0.154	0.469	Aldrich	53675
Viridiflorene	y=0.034x-0.001	0.999	11.53-1.73	5.07	0.501	1.522	Aldrich	61770

Each internal standard is the reference compound for the analytes that follow. [a]Response ratio vs. concentration ratio, internal standard correction applied. [b]Quantifier ion. RSD = Relative standard deviation. LOD = Limit of detection (signal/noise = 3). LOQ = Limit of quantitation (signal/noise = 10).
